# Clinical Value and Underlying Mechanisms of Upregulated *LINC00485* in Hepatocellular Carcinoma

**DOI:** 10.3389/fonc.2021.654424

**Published:** 2021-07-05

**Authors:** Xinyu Zhu, Yanlin Feng, Dingdong He, Zi Wang, Fangfang Huang, Jiancheng Tu

**Affiliations:** Department of Laboratory Medicine, Clinical Laboratory Medicine and Center for Gene Diagnosis, Zhongnan Hospital of Wuhan University, Wuhan, China

**Keywords:** long non-coding RNA, hepatocellular carcinoma, prognosis, diagnosis, biomarker, ceRNA

## Abstract

**Aims:**

This study aimed to reveal the functional role of *LINC00485* in hepatocellular carcinoma (HCC).

**Materials & Methods:**

210 serum samples from Zhongnan Hospital of Wuhan University were employed to evaluate clinical value of *LINC00485*. Bioinformatics analysis was adopted to explore its potential mechanisms.

**Results:**

*LINC00485* was confirmed to be upregulated in HCC tissues and serum samples. Survival analysis and receiver operating characteristic curve revealed its prognostic and diagnostic roles. The combination of serum *LINC00485* with AFP can remarkably improve diagnostic ability of HCC. Exploration of the underlying mechanism demonstrated that *LINC00485* might exert pro-oncogenic activity by *LINC00485*—three miRNAs—four mRNAs network.

**Conclusions:**

Our study unveiled that upregulated *LINC00485* could act as a potential diagnostic and prognostic biomarker and provide a novel insight into the molecular mechanisms of *LINC00485* in HCC pathogenesis.

## Introduction

On a Global scale, about 782,000 deaths are attributable to primary liver cancer (PLC) and 841,000 are newly diagnosed every year (1). Hepatocellular carcinoma (HCC), one of the most common malignant tumors and the main pathological type of PLC (accounting for 85%-90%) (2), ranks third in cancer-related lethality and sixth among cancers in terms of incidence. Particularly, more than 50% of HCC cases are observed in East Asia and parts of Africa (3). Due to the concealed onset and absence of effective screening strategies, approximately 80% of HCC patients are prone to be diagnosed at intermediate-advanced stage, causing poor prognosis with 5-year survival of only 12% in China (4). In the clinic, Serological markers is one of the diagnostic tools for HCC and alpha fetoprotein (AFP) is the main tumor biomarker available to guide the management of HCC (5). However, studies in recent years have indicated that about 30%-40% of overall HCC patients have normal AFP levels. Thus, the development of screening more accurate non-invasive biomarkers for early detection of HCC is urgently required (6).

Long non-coding RNAs (lncRNAs) are a class of RNA transcripts that lack significant protein-coding function with the length of sequence more than 200 nucleotides (7). Accumulating evidence suggests that the occurrence of many human cancers is closely connected with lncRNAs abnormal expression or dysfunction. With advances in genomics and bioinformatics, lncRNAs have been proved to be participated in the regulation of numerous important biological processes, such as transcriptional activation and interference, chromatin modification, intracellular signaling, cell cycle and so on (8). Meanwhile, growing studies support that lncRNAs can act as competing endogenous RNAs (ceRNAs) which could competitively bind to microRNAs (miRNAs), resulting in the dysregulation of miRNAs and subsequently the aberrant expression of corresponding target mRNAs (9). Notably, many studies have indicated that ceRNA networks play an important role in the tumorigenesis and cancer progression of HCC, including cancer cell growth, epithelial to mesenchymal transition (EMT), metastasis and chemoresistance (10, 11). For example, lncRNA AGAP2-AS1 upregulates ANXA11 expression by sponging miR-16-5p and promotes proliferation and metastasis in HCC (12); lncRNA DANCR promotes HCC progression and regulates EMT by sponging miR-27a-3p *via* ROCK1/LIMK1/COFILIN1 pathway (13). However, till date, many lncRNAs have not been investigated for its role in HCC.

**Figure 1:**
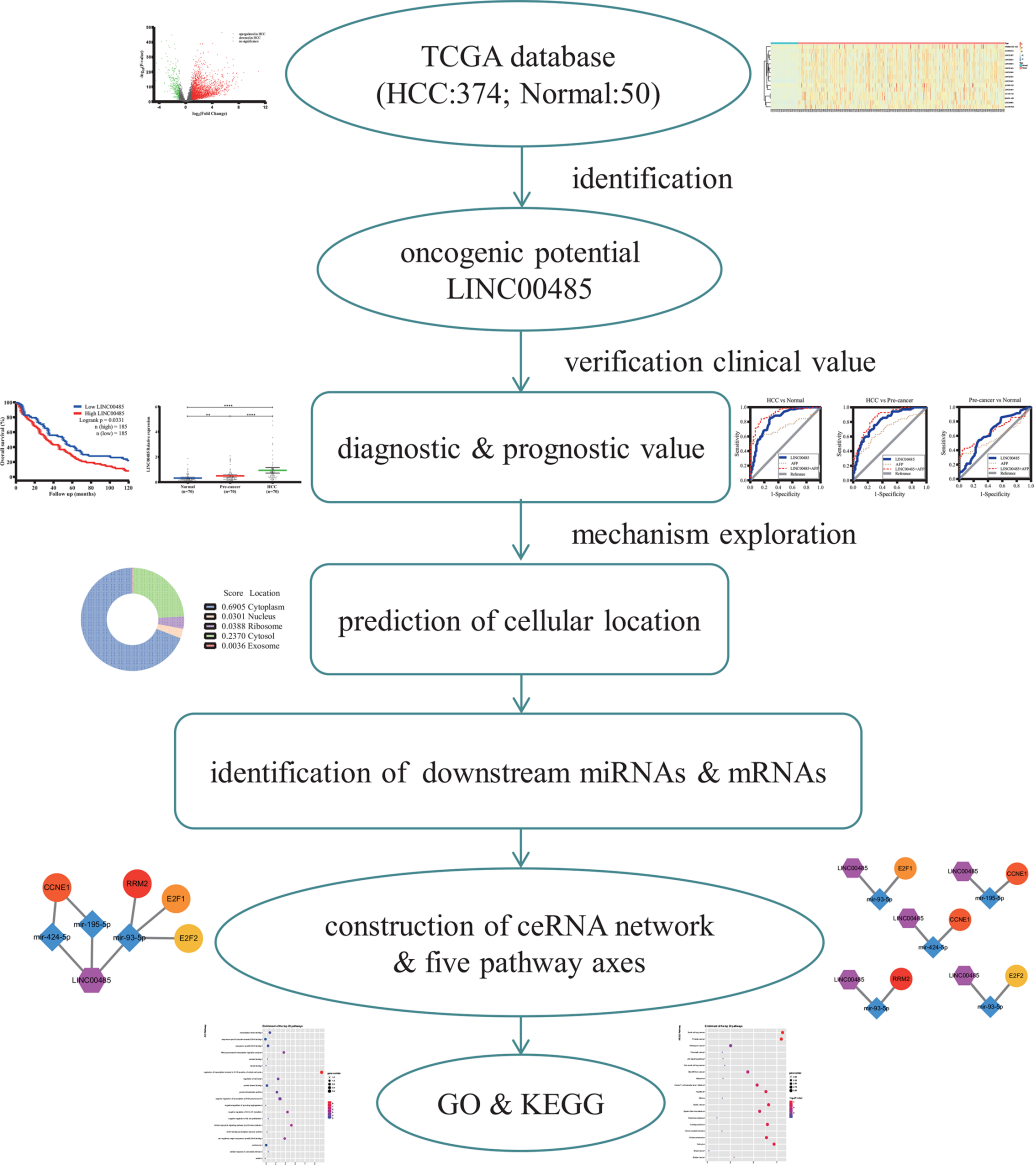
The flow diagram of the entire work. TCGA, The Cancer Genome Atlas; GO, Gene oncology; KEGG, Kyoto Encyclopedia of Gene and Genome.

In this paper, we aimed to investigate the role of *LINC00485* in HCC and explore its underlying regulatory mechanisms by combining real-time PCR and bioinformatics analysis. An overview flow diagram of the study is depicted in **Figure 1**. Firstly, we downloaded gene expression data from TCGA database and acquired differentially expressed LncRNAs *via* R language software, among which we selected *LINC00485* and evaluated its prognostic and diagnostic performance after analyzed its expression in HCC serum samples. Then we used online tools to identify its downstream targets and potential biological mechanism involved in HCC. Ultimately, we constructed lncRNA**—**miRNA**—**mRNA network and protein**—**protein interaction (PPI) followed by functional enrichment analysis. This work is the first to explore *LINC00485* as a novel biomarker and might offer some new reliable insights into HCC pathogenesis.

## Materials and Methods

### Screen for Potential lncRNAs

Gene expression data of human HCC, relative to 370 tumor samples and 50 normal samples, were downloaded from the Cancer Genome Atlas (TCGA) database, and analyzed *via* the R language software. Gene expression data of the TCGA were normalized and screened for differentially expressed lncRNAs using edgeR and limmaR package, adjusted p value (FDR) < 0.05 and the threshold of |log2(foldchange)| >1 is considered as the screening criteria. Heatmap and volcano plot were depicted using R software version 4.0.2.

### Patients Data and Serum Samples

Serum samples were collected from 70 patients with hepatitis or liver cirrhosis (defined as pre-cancer), 70 patients with HCC and 70 age-matched healthy persons admitted to Zhongnan Hospital of Wuhan University (Wuhan, China) between December 2019 and September 2020, all patients have been pathologically and histologically confirmed according to their reports. The collected samples were centrifuged at 12,000 rpm for 15 min at 4°C, then, the serum supernatant was carefully transferred into RNase-free tube and stored at -80°C for further analyses. Our study was approved by the Ethics Committee of Zhongnan Hospital of Wuhan University.

### RNA Extraction and Quantification

Total RNA in serum was extracted using RNA Separate Extraction Kit (Bioteke, Beijing, China) under the manufacturer’s protocol. The concentration and purity of total RNA was assessed with a NanoDrop 2000 (Thermo, USA), and then reverse-transcribed into complementary DNA (cDNA) using the PrimeScript™ RT reagent kit with gDNA Eraser (Takara, Japan). Real-time PCR was performed on the Bio-Rad CFX96 (Bio-Rad, Hercules, CA, USA) using Ultra-SYBR mixture (CWBIO, China) in order to measure lncRNA levels. The Glyceraldehyde-3-phosphate dehydrogenase (GAPDH) was selected as the endogenous control. Each sample was detected twice simultaneously and 2^-(Ct^ *^LINC00485^* ^-Ct GAPDH)^ was used to calculate the relative expression. The PCR procedure included a hot start step of 5 min at 95°C followed by 40 cycles of amplification, each cycle consisted of denaturation at 95°C for 30 sec, annealing at 61.6°C for 30 sec and elongation at 72°C for 30 sec. Primer Sequences are listed in **Table S1**.

### Prognostic and Diagnostic Value of *LINC00485*

To evaluate the prognostic effect of *LINC00485* in HCC, clinical prognostic information of 370 HCC patients involving follow-up time and survival condition were acquired from the TCGA database. According to the median level of *LINC00485*, patients were separated into two subgroups (Low *LINC00485* group and High *LINC00485* group). To further identify its diagnostic value in distinguishing HCC patients, the receiver operating characteristic (ROC) curve was performed by using three models (HCC versus Pre-cancer, HCC versus Normal, Pre-cancer versus Normal). Area under the curve (AUC) was used to assess the diagnostic performance of serum *LINC00485*.

### Prediction of *LINC00485* Cellular Localization

We used LncLocator (http://www.csbio.sjtu.edu.cn/bioinf/lncLocator/) to predict *LINC00485* cellular location (14), it’s a website that predicts the subcellular location of lncRNA based on sequence (FASTA file) (**Table S2**).

### Prediction of Downstream miRNAs

MiRcode (http://www.mircode.org/index.php) provides microRNA target prediction based on the comprehensive GENCODE gene annotation. The potential target miRNAs of *LINC00485* were obtained from miRcode. Then, to further verify these target miRNAs expression level in human HCC, miRNAmatrix data of liver hepatocellular carcinoma (LIHC) were downloaded from the TCGA database. The differentially expressed miRNAs were defined with FDR < 0.05 and |log2(foldchange)| >1. Only those differentially expressed in TCGA-LIHC were considered as the potential downstream miRNAs of *LINC00485*.

### Analysis of lncRNA—miRNA Correlation

To further confirm the correlation between *LINC00485* and target miRNAs, we evaluated their expression levels in the same patients from TCGA-LIHC. P value in Pearson’s correlation test less than 0.05 was considered to have statistical significance. Then, we performed meta-analysis to verify the expression of target miRNAs in HCC based on nine datasets. We extracted useful information comprising original miRNA expression data, dataset number, platform number, first author, country, published date and number of patients from selected datasets. Stata SE12.0 (STATA Corp, College Station, Texas, USA) was used to calculate the pooled standard mean difference (SMD) and its 95% confidence internal (CI). Pooled result not equal to 0 indicating that this miRNA is meaningful in HCC patients. DIANA miRPath was used to predict the significantly correlated pathways of target miRNAs.

### Prediction of Downstream mRNAs and PPI Network and Hub Genes

MiRDB (http://mirdb.org/), miRTarBase (http://mirtarbase.mbc.nctu.edu.tw/php/index.php) and TargetScan (http://www.targetscan.org/vert_72/) were used to predict miRNA target genes. Only mRNAs predicted by all three databases were identified as candidate mRNAs. mRNAmatrix data were downloaded from TCGA-LIHC to analyze target genes expression levels. We exclusively chose genes that differentially expressed in TCGA-LIHC as the relevant downstream mRNAs. Accordingly, the preliminary interaction network of lncRNA**—**miRNA**—**mRNA was constructed and visualized by Cytoscape v3.7.2 software. The protein**—**protein interaction network was derived from STRING v11.0 (http://string-db.org/) and Cytoscape was used for visualization. CytoHubba, a plugin of Cytoscape, was applied to screen the top ten hub genes.

### Verification of lncRNA—miRNA—mRNA Network

Correlation analysis, survival analysis and protein expression were employed to establish ceRNA network. The interaction between *LINC00485* and mRNA as well as miRNA and mRNA were investigated by assessing their corresponding expression levels in the same patients (TCGA-LIHC). Survival analysis was conducted to validate the effect of ten hub genes in HCC patients’ disease-free survival (DFS) and overall survival (OS) through GEPIA database (http://gepia.cancer-pku.cn/). We used the Human Protein Atlas (HPA, https://www.proteinatlas.org/) to evaluate hub genes’ protein expression in healthy liver and HCC tissues based on immunohistochemistry (IHC) data (15). All the information of the antibodies used in IHC assay were listed in **Table S3**. For each specimen, the proportion of stained cells (< 25%, 25-75% or > 75%) and the staining intensity (negative, weak, medium or strong) were first assessed by two experts. The expression score was then divided into four subtypes determined by the combination of stained cells proportion and staining intensity: not detected (1-negative, 2-<25% combined with weak); low (1-either 25-75% or >75% combined with weak, 2-<25% combined with moderate); medium (1-either 25-75% or >75% combined with moderate, 2-<25% combined with strong); high (1-either 25-75% or >75% combined with strong). If the expression score was “not detected”, the specimen was labeled as negative. And if the expression score was ‘low’, ‘medium’ or ‘high’, the specimen was labeled as positive. The expression score reflects the level of the gene in this specimen.

### GO and KEGG Analysis

KEGG Orthology Based Annotation System (KOBAS, http://kobas.cbi.pku.edu.cn/kobas3) was used to execute Gene Ontology (GO) and Kyoto Encyclopedia of Genes and Genomes (KEGG) pathway analysis for the hub genes. It’s one of the most widely used web servers for gene/protein functional annotation and gene set enrichment.

### Statistical Analysis

All statistical analyses were done in GraphPad Prism v8.0 (GraphPad Software, CA, USA) and IBM SPSS Statistics v26.0 (SPSS, Inc., IL, USA). Kolmogorov Smirnov test were applied to check for normality distribution of variables. Means were compared by independent sample T-tests. Multiple group comparisons were performed by one-way analysis of variance (ANOVA) when the variances are homogeneous. Kruskal-Wallis H test was conducted to judge any significant differences of non-normally distributed variables. Chi-square (χ2) test was used to assess categorical variables. For all analyses, statistical significance was accepted at P <0.05.

## Results

### Differentially Expressed lncRNAs in HCC

**Figure 2:**
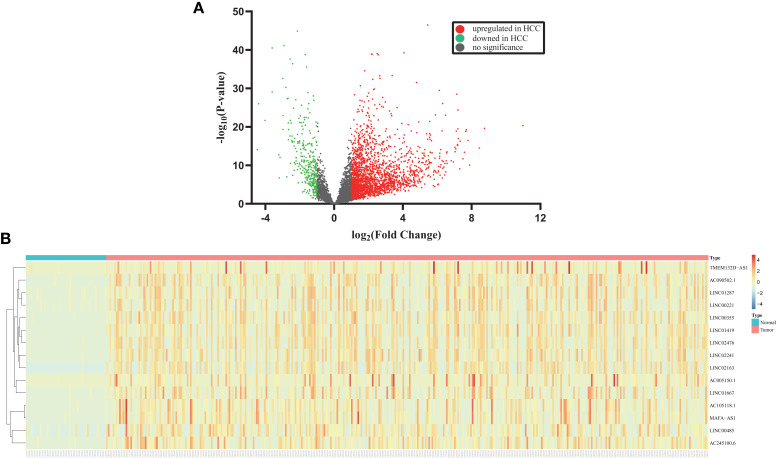
Identification of differentially expressed lncRNAs in HCC from TCGA. **(A)** Volcano plot displays the changes of lncRNA expression profiles in the TCGA. Each dot represents a lncRNA. Red and green dots indicate upregulated and downregulated lncRNAs, respectively. **(B)** Heatmap shows the detailed expression patterns of fifteen dysregulated lncRNAs in TCGA samples. Each colored block at different position reflects the relative expression of corresponding lncRNA in relevant sample. Red indicates high expression and blue indicates low expression.

As a result, 2626 differentially expressed lncRNAs were identified from TCGA-LIHC based on adjusted p value (FDR) < 0.05 and the threshold of |log2(foldchange)| >1, whereas 2296 lncRNAs were upregulated and 330 lncRNAs were downregulated. As shown in **Figure 2A**, the volcano plot displayed gene expression differences between the healthy and HCC groups. The heatmap were employed to demonstrate the expression of fifteen dysregulated lncRNAs including *LINC00485* (**Figure 2B**).

### Prognostic Value of *LINC00485* in HCC Tissues

**Figure 3:**
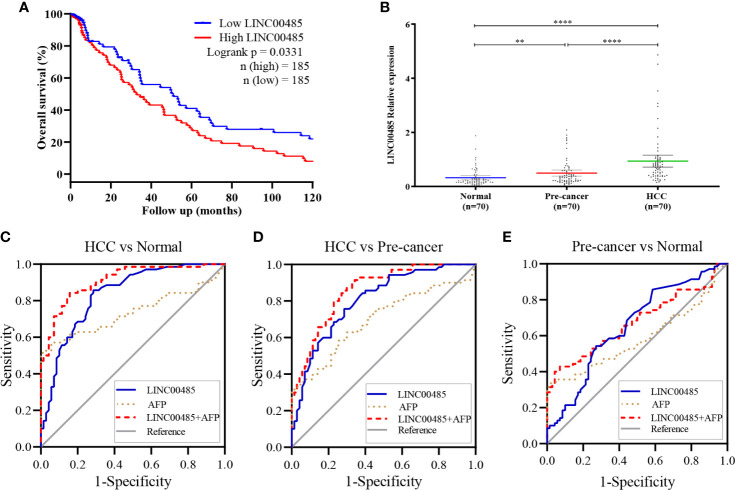
Validation of *LINC00485* expression in HCC serum and its prognostic & diagnostic performance. **(A)** Survival analysis shows the relationship between expression levels of *LINC00485* and overall survival of patients with HCC. The X axis represents follow-up time (months) and the Y axis represents survival rate. Different colored curve reflects different groups, red reflects high *LINC00485* group, and blue reflects low *LINC00485* group. **(B)** Scatter plot shows the differences between serum expression of *LINC00485* in HCC, pre-cancer and normal controls detected by real-time PCR method. **p < 0.01, ****p < 0.0001. ROC curve reflects the diagnostic value of *LINC00485* and AFP in discriminating: **(C)** HCC from Normal, **(D)** HCC from Pre-cancer, and **(E)** Pre-cancer from Normal. ROC, Receiver operating characteristic. Area under the curve (AUC) represents the diagnostic efficiency.

We selected *LINC00485* for subsequent analysis since previous studies have not explored the effect of significantly upregulated *LINC00485* in HCC. Then, we combined the expression of *LINC00485* with the clinical prognostic data of 370 HCC patients to assess the prognostic value of *LINC00485*. Kaplan-Meier survival curve exhibited that LINC00485 levels in tissues were correlated with patients OS, patients with high *LINC00485* had a worse OS compared with low *LINC00485* group (median survival time, 32.83 months and 51 months, respectively; p = 0.0331) **(** **Figure 3A**). Thus, *LINC00485* might be considered a critical factor for poor prognosis of HCC.

### Diagnostic Value of *LINC00485* in HCC Serum

Results showed significantly higher levels of serum *LINC00485* in HCC patients compared with pre-cancer patients (p < 0.0001) and healthy individuals (p < 0.0001) (**Figure 3B**). **Table 1** detailed the clinical information of enrolled participants. Then to determine whether *LINC00485* could be used as a noninvasive biomarker for HCC diagnosis, we conducted ROC curve analysis. Results demonstrated that *LINC00485* has a significant diagnostic effect in distinguishing HCC from normal persons (AUC = 0.8336, sensitivity: 85.71%, specificity: 71.42%, 95% CI: 0.7667 - 0.9004, p < 0.0001). Meanwhile, *LINC00485* could act a predictive role in differentiating pre-cancer from HCC and normal individuals (HCC vs pre-cancer: AUC = 0.8039, sensitivity: 75.71%, specificity: 71.43%, 95% CI: 0.7322–0.8755, p < 0.0001; pre-cancer vs normal: AUC = 0.6551, sensitivity: 54.29%, specificity: 72.86%, 95% CI: 0.5645–0.7457, p = 0.0015) (**Table 2** and **Figures 3D, E**). Then we combined *LINC00485* with AFP, and speculated that the combination may increase the detection rate of HCC, our result also evidenced this, The AUC of *LINC00485* combined with AFP was greater than that of AFP **(** **Figures 3C–E**). Together, these results supported the feasibility and diagnostic value of serum *LINC00485* in HCC.

**Table 1:** Clinical characteristics of patients in the study.

Characteristics	HCC (n = 70)	Pre-cance r(n = 70)	Normal (n = 70)	p-value
Gender				0.851^£^
Female (%)	19 (27.1)	22 (31.4)	20 (28.6)	
Male (%)	51 (72.9)	48 (68.6)	50 (71.4)	
Age (y)				0.216^£^
< 55 (%)	32 (45.7)	39 (55.7)	30 (42.9)	
≥ 55 (%)	38 (54.3)	31 (44.3)	40 (57.1)	
ALT (U/L)^†^	34 (19 - 66)	28.5 (17 - 66.5)	21 (18.25 - 30)	<0.001^§^
AST (U/L)^†^	61 (27.25 - 120)	50 (28 - 90.25)	20 (17 - 23)	<0.001^§^
TBIL (μmol/L)^†^	20.1(15 – 28.3)	49.9 (30.7 - 131.5)	15 (11.9 – 18.2)	<0.001^§^
DBIL (μmol/L)^†^	4.8 (3.12 - 6.87)	16.1 (7.9 – 65.21)	2.9 (2.61 – 4.01)	<0.001^§^
TP (g/L)^†^	65.9 (61.97 - 75.21)	60.07 (52.45 - 68.13)	72.1 (69.1 - 75.4)	<0.001^§^
ALB (g/L)^†^	37 (30.8 - 39.29)	29.8 (28 - 37.1)	42.9 (42.1 – 45.1)	<0.001^§^
GGT (U/L)^†^	72 (39.5 - 179.4)	49 (23.1 – 101.6)	25 (20.4 – 40.8)	<0.001^§^
ALP (U/L)^†^	130 (79.5 - 220)	107 (79.4 – 160.8)	64 (49 - 81)	<0.001^§^
TBA (μmol/L)^†^	12.4 (3.48 - 24.71)	40.5 (9.6 - 151.24)	2.81 (1.5 – 5.7)	<0.001^§^
AFP (ng/mL)^†^	62.8 (2.12 - 1021.1)	3.93 (1.15 - 60.38)	3.01 (1.35 - 5.08)	<0.001^§^
CEA (ng/mL)^†^	2.19 (1.49 - 2.86)	2.36 (1.27 - 3.55)	1.4 (0.89 - 2.14)	<0.001^§^

^†^median (first quartile – third quartile); ^§^Kruskal–Wallis test; ^£^χ^2^ test; ALT, Alanine aminotransferase; AST, Aspartate aminotransferase; TBIL, Total bilirubin; DBIL, Direct bilirubin; TP, Total protein; ALB, Albumin; GGT, Gammaglutamyl transpeptidase; ALP, Alkaline phosphatase; TBA, Total bile acid; AFP, Alpha-fetoprotein; CEA, Carcino-embryonic antigen.

**Table 2:** Comparisons of the diagnostic value of *LINC00485*, AFP and AFP combined *LINC00485* in three models.

Model	Biomarker	AUC	95% CI	p-value	Se (%)	Sp (%)
HCC vs Normal	*LINC00485*	0.8336	0.7667-0.9004	<0.0001	85.71	71.42
	AFP	0.7360	0.6485-0.8235	<0.0001	51.43	100
	AFP+*LINC00485*	0.9098	0.8629-0.9567	<0.0001	84.29	84.29
HCC vs Pre-cancer	*LINC00485*	0.8039	0.7322-0.8755	<0.0001	75.71	71.43
	AFP	0.6968	0.6088-0.7849	<0.0001	62.86	70
	AFP+*LINC00485*	0.8571	0.7969-0.9174	<0.0001	92.86	64.29
Pre-cancer vs Normal	*LINC00485*	0.6551	0.5645-0.7457	0.0015	54.29	72.86
	AFP	0.5844	0.4870-0.6817	0.0848	31.43	98.57
	AFP+*LINC00485*	0.6749	0.5847-0.7651	0.0004	42.86	92.86

AUC, Area under the receiver operating characteristic curves; Se, Sensitivity; Sp, Specificity.

### Analysis of lncRNA—miRNA Interactions

**Figure 4:**
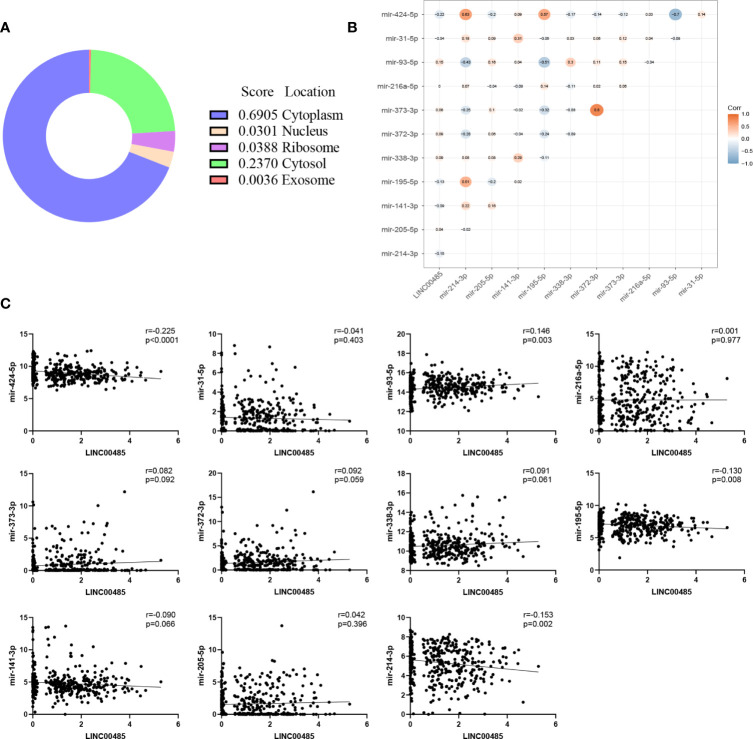
The cellular localization of *LINC00485*. **(A)** Subcellular location of *LINC00485* predicted by LncLocator. The location is determined by the score of different subcellular components. Correlation analysis between *LINC00485* and downstream miRNAs. **(B)** Heatmap of the correlation between expression levels of *LINC00485*, miR-214-3p, miR-205-5p, miR-141-3p, miR-195-5p, miR-338-3p, miR-372-3p, miR-373-3p, miR-216a-5p, miR-93-5p, miR-31-5p, and miR-424-5p in the same HCC patients derived from TCGA database. Pearson correlation was applied to explore the relevance. The colored dots at different positions reflect the correlation between the corresponding molecules. The red dots indicate positive correlation while the blue dots indicate negative correlation. **(C)** Each scatter plot represents the correlation between *LINC00485* and each downstream miRNA.

Data from LncLocator showed that *LINC00485* was mainly localized in the cytoplasm, suggesting that it might act as a sponge of miRNAs (**Figure 4A**). Our results indicated that thirty miRNAs may interact with *LINC00485*, of which only eleven miRNAs were differentially expressed in HCC (FDR < 0.05 and |log2foldchange| >1). Then after executing the Pearson’s correlation test, we found that only four miRNAs were considered to be correlated with *LINC00485* (P< 0.05) (**Figures 4B, C**). So far, the lncRNA—miRNA interactions included *LINC00485* and three downregulated miRNAs (miR-214-3p, miR-195-5p, miR-424-5p) and one upregulated miRNA (miR-93-5p).

**Figure 5:**
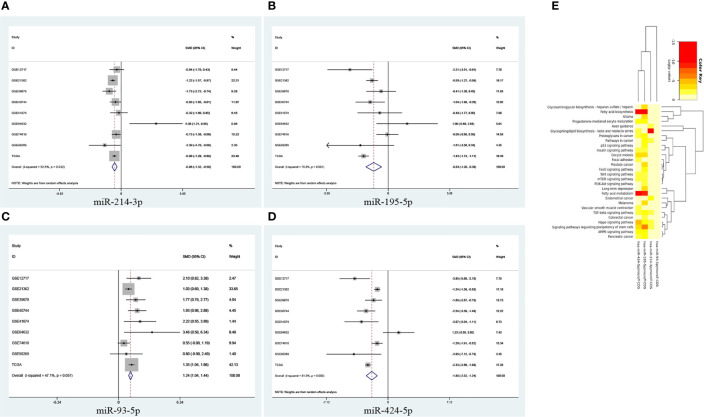
Forest diagrams of datasets assessing the levels of the predicted miRNAs in HCC: **(A)** miR-214-3p, **(B)** miR-195-5p, **(C)** miR-93-5p, **(D)** miR-424-5p. **(E)** Heatmap for the signal pathways from DIANA−miRPath in which the four miRNAs are involved.

We adopted nine datasets to validate the expression level of four miRNAs in HCC. The essential features of the nine datasets were listed in **Figure S1**. Pooled results proved that there was a significant difference in the expression level of the four miRNAs between the HCC and normal groups. (miR-214-3p: SMD = -0.96, 95% CI = -1.32 ~ -0.59; miR-195-5p: SMD = -0.83, 95% CI = -1.29 ~ -0.38; miR-93-5p: SMD = 1.24, 95% CI = 1.04 ~ 1.44; miR-424-5p: SMD = -1.88, 95% CI = -2.52 ~ -1.24) (**Figures 5A–D**). For subsequent analysis, we used DIANA miRPath to show the significantly correlated pathways of the four miRNAs. From **Figure 5E**, we found that these four miRNAs predominantly participated in Fatty acid metabolism and cancer-related signaling pathways such as Hippo signaling pathway.

### LncRNA—miRNA—mRNA Network Construction

**Figure 6:**
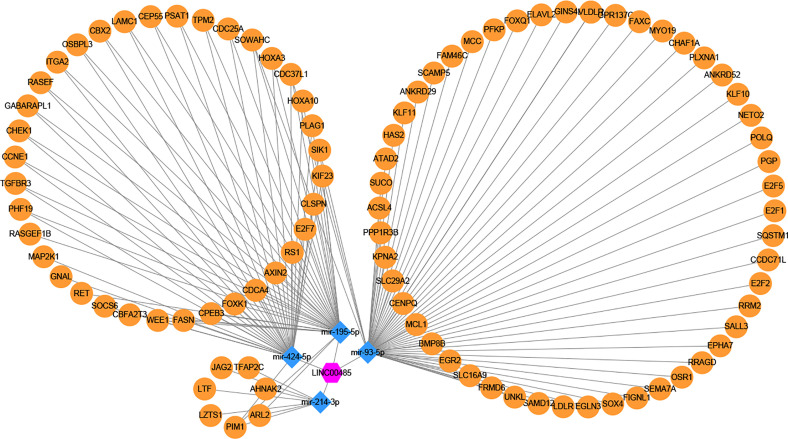
Construction of lncRNA–miRNA–mRNA regulatory network. One candidate lncRNA (purple hexagon), four candidate miRNAs (blue diamond) and ninety-four potential downstream mRNAs (yellow ellipse) form the network. The candidate miRNAs of *LINC00485* were predicted by miRcode and verified by correlation analysis and meta-analysis. The potential downstream mRNAs of candidate miRNAs were retrieved from miRDB, miRTarBase and TargetScan. The regulatory network of lncRNA–miRNA–mRNA was constructed and visualized by Cytoscape. Edges represent the interactions between these RNAs.

Three online tools (miRDB, miRTarBase and TargetScan) were used to predict the potential target genes of these four miRNAs, and 692 target genes were found, among which only ninety-four genes were differentially expressed. Then, the miRNA**—**mRNA interactions involving four miRNAs and ninety-four mRNAs were constructed. The interaction pairs of lncRNA**—**miRNA and miRNA**—**mRNA were combined to establish a complete lncRNA**—**miRNA**—**mRNA network, which consisted of one lncRNA (*LINC00485*), four miRNAs (miR-214-3p, miR-195-5p, miR-93-5p, miR-424-5p) and ninety-four mRNAs (**Figure 6**).

### Identification of Hub Genes From PPI Network

**Figure 7:**
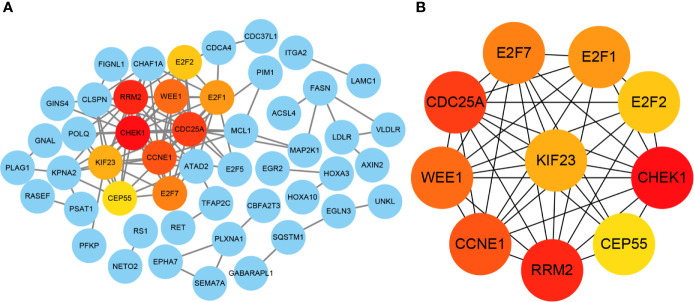
Identification of hub genes from the PPI network with CytoHubba. **(A)** Visualization of PPI network. The network was established by STRING and visualized by Cytoscape. Each circle represents each potential downstream mRNA, and the lines represent the interactions among these mRNAs. Hub genes with the most interactions are highlighted in the current PPI network. **(B)** Correlation between the hub genes. PPI, Protein-protein interaction.

The ninety-four mRNAs were entered into STRING database for PPI network construction and Cytoscape was used for visualization (**Figure 7A**). The top ten hub genes were WEE1, RRM2, KIF23, E2F7, E2F2, E2F1, CHEK1, CEP55, CDC25A, CCNE1, which were determined from the PPI network using the CytoHubba plugin (**Figure 7B**). After ten key genes were identified, we found that one of the four miRNAs did not interact with the ten key genes(miR-214-3p). So far, the current lncRNA—miRNA—mRNA network includes *LINC00485*, three miRNAs (miR-195-5p, miR-93-5p, miR-424-5p) and ten mRNAs (WEE1, RRM2, KIF23, E2F7, E2F2, E2F1, CHEK1, CEP55, CDC25A, CCNE1). This network illustrates the primary regulatory mechanism of *LINC00485* in HCC.

### Construction of ceRNA Network

**Figure 8:**
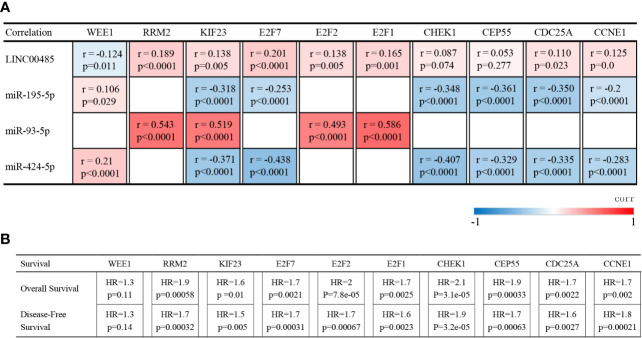
Verification of lncRNA–mRNA and miRNA–mRNA pairs. **(A)** Correlation between expression levels of *LINC00485*, miR-195-5p, miR-93-5p, miR-424-5p, WEE1, RRM2, KIF23, E2F7, E2F2, E2F1, CHEK1, CEP55, CDC25A and CCNE1 in the same HCC patients derived from TCGA database. Pearson correlation was applied to explore the relevance. The colored blocks at different positions reflect the level of correlation between the corresponding molecules. **(B)** Impact of hub genes expression on HCC patients’ overall survival and disease-free survival analyzed by GEPIA. HR: HR (high).

**Figure 9:**
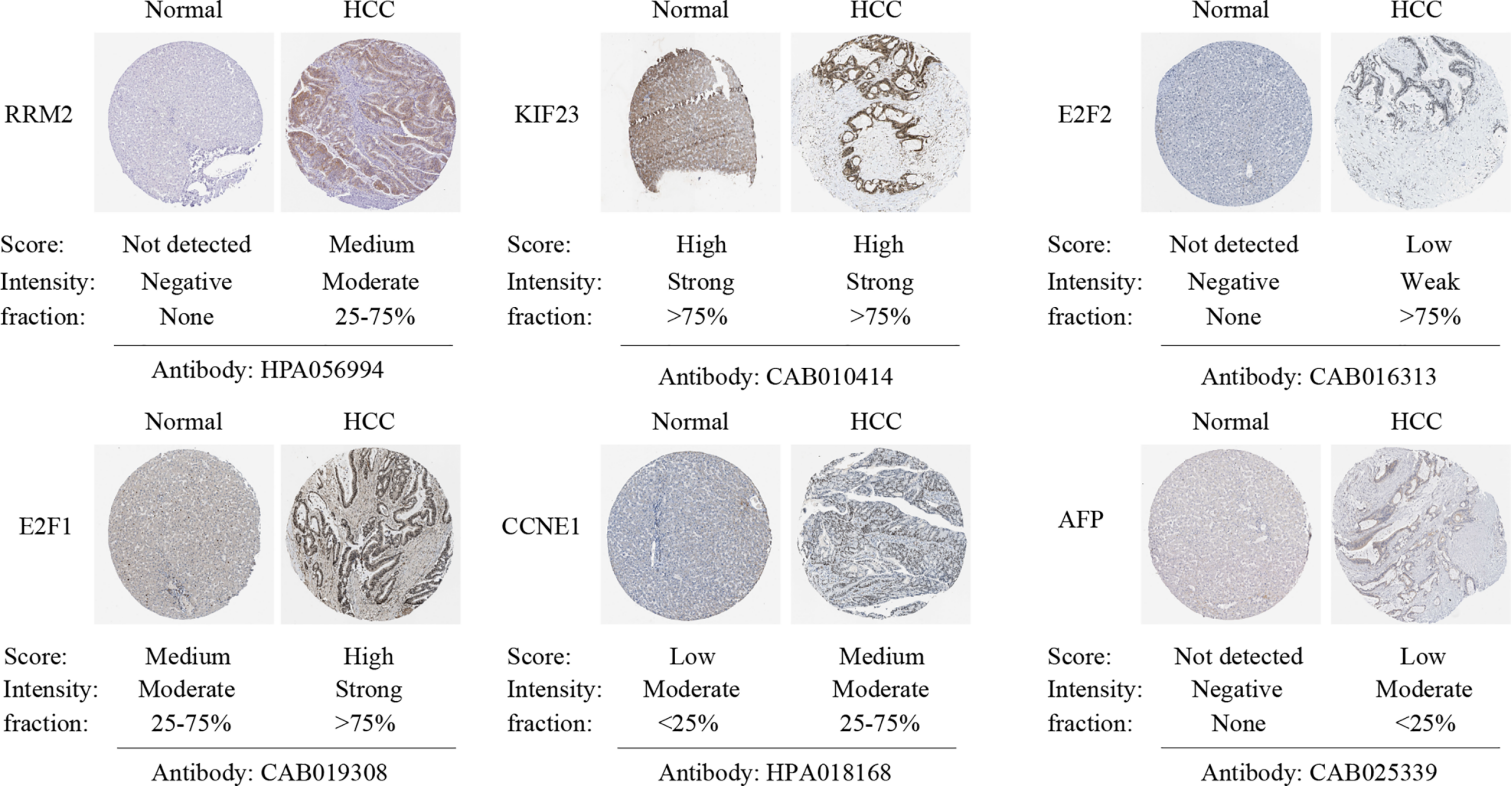
Verification of hub genes protein expression in HCC and normal liver tissues. Immunohistochemistry images of hub genes and AFP in HCC and normal liver tissues based on Human Protein Atlas (HPA). The expression score is categorized into high, medium, low, and not detected. The expression score reflects the protein level of the gene in this specimen.

The correlation between *LINC00485*, three miRNAs and ten hub genes were summarized in **Figure 8A**. Surprisingly, correlations were found in almost all of these pairs, except *LINC00485*/CHEK1 and *LINC00485*/CEP55 (P > 0.05) (**Figures S2**, **S3**). Moreover, Survival analysis further indicated that seven hub genes (RRM2, KIF23, E2F7, E2F2, E2F1, CDC25A, CCNE1) had impact on HCC patients’ poor OS and DFS except WEE1 (P > 0.05) (**Figures 8B** and **S4**). To further narrow down the gene set, HPA data were used to observe abnormal protein expression. Due to the absence of data on E2F7 and CDC25A genes in the HPA database, we just analyzed the protein expression of the remaining five hub genes. (**Figure 9**). To ensure the reliability of our results, we also investigated AFP levels in HCC and healthy liver tissue. After comparing the expression score of HCC tissue and normal tissue, we found that the level of AFP in normal tissue was lower than that of HCC tissue, indicating the quality of the sample is quite high. HPA results showed that the protein levels of RRM2, E2F2, E2F1 and CCNE1 were highly expressed in HCC, whereas there was no difference in the expression of KIF23. From the above results, we identified four highly expressed key genes in HCC tissues, namely RRM2, E2F2, E2F1 and CCNE1, which interact with *LINC00485* and also exhibit prognostic value in HCC.

**Figure 10:**
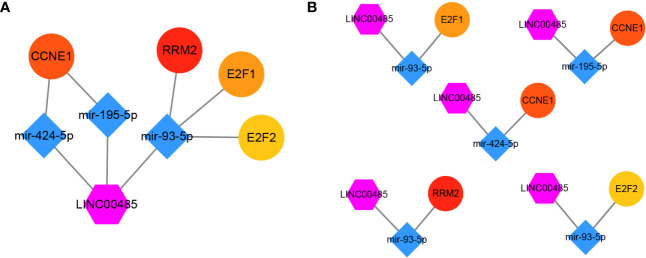
Visualization of the main network. **(A)** One candidate lncRNA (purple hexagon), three candidate miRNAs (blue diamond) and four candidate mRNAs (ellipse) form the main network. **(B)** five possible pathway axes identified from the network.

Based on the results of our study, the final lncRNA**—**miRNA**—**mRNA network was formed including one lncRNA, three miRNAs, and four hub genes (**Figure 10A**). Five lncRNA–miRNA–mRNA regulatory pathways (axes), namely *LINC00485*/miR-424-5p/CCNE1, *LINC00485*/miR-195-5p/CCNE1, *LINC00485*/miR-93-5p/E2F1, *LINC00485*/miR-93-5p/E2F2 and *LINC00485*/miR-93-5p/RRM2 were then identified from the network (**Figure 10B**).

### Functional Enrichment of the Four Hub Genes

**Figure 11:**
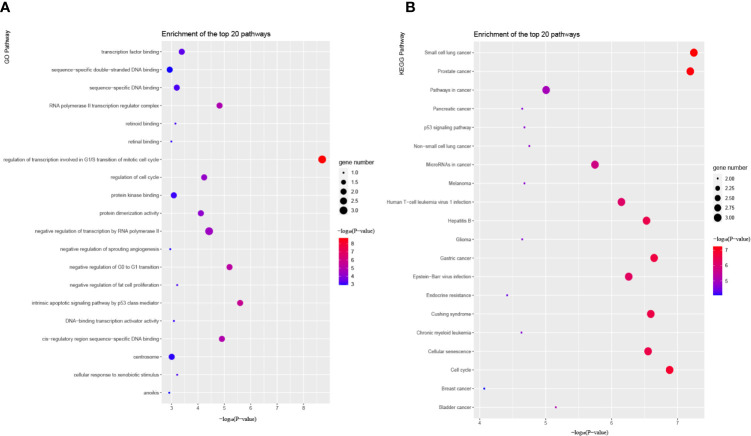
Functional enrichment analysis of candidate hub genes in the network. **(A)** Bubble plot of GO pathways. **(B)** Bubble plot of KEGG pathways. GO and KEGG pathway analysis show the potential functions of candidate hub genes.

To gain a better understanding of the four hub genes, we performed GO and KEGG pathway enrichment in KOBAS. According to GO term annotation (**Figure 11A**), it suggested that these four mRNAs may be involved in modulating some biological processes closely related to human cancers, such as ‘Transcription factor binding’, ‘Regulation of cell cycle’, ‘negative regulation of transcription by RNA polymerase II’, ‘intrinsic apoptotic signaling pathway by p53 class mediator’ and ‘regulation of transcription involved in G1/S transition of mitotic cell cycle’ processes. Additionally, Enrichment analysis for KEGG pathways showed that these mRNAs are also participated in regulating some cancer-related pathways, such as ‘MicroRNAs in cancer’, ‘Cellular senescence’ and ‘Hepatitis B’ pathways (**Figure 11B**). Together, the results of the functional enrichment analysis revealed that the five regulatory pathways might play a pivotal role in the progression of HCC.

## Discussion

To systematically identify more significant lncRNAs that might be potentially involved in the pathogenesis of HCC, we downloaded lncRNA expression profiles from TCGA database for subsequent research. Among all the differentially expressed lncRNAs, the upregulation of *LINC00485* in tumor tissues attracted our attention. For a long time, its carcinogenic effect has not been emphasized. Until recently, it was reported by Zhang et al. that *LINC00485* promotes lung cancer progression by modulating miR-298/c-Myc axis (16). However, the specific mechanism of *LINC00485* in the development of HCC has not been delineated. Therefore, in the present study, we evaluated its prognostic and diagnostic value and attempted to reveal the underlying regulatory pathways in HCC.

First of all, we detected the serum expression levels of *LINC00485* in HCC patients, pre-cancer patients and healthy people by real time-PCR. The results demonstrated that the level of *LINC00485* in HCC was significantly higher than that in precancerous and normal person, which was consistent with the data obtained from the TCGA. ROC curve analyses further illustrated that serum *LINC00485* had an acceptable diagnostic value for distinguishing HCC from precancerous and healthy person. Besides, we also found that its diagnostic efficiency was somewhat superior to AFP. Nowadays, Ultrasonography and AFP detection are commonly accepted as useful methods to periodically screen HCC (17–19). Imaging diagnosis of early HCC still remains some troubles, and serum AFP has a non-negligible false negative rate of about 30%, which could potentially lead to misdiagnosis, missed diagnosis, and a delay in timely treatment (20–22). Therefore, we planned to analyze whether *LINC00485* can help clinicians better distinguish HCC patients. ROC curve analyses indicated that combined detection of *LINC00485* and AFP had a better diagnostic value than that of single marker for the discrimination of HCC from healthy person. Additionally, Kaplan-Meier survival plot showed that HCC patients with high expression levels of *LINC00485* manifested shorter survival time and poor prognosis. The above results indicated that *LINC00485* may be a potential diagnostic and prognostic indicator of HCC.

Understanding the molecular mechanisms that underlie tumor biology is of the utmost importance if therapeutic targets are to be identified. Therefore, exploring different regulatory pathways of *LINC00485* in HCC will provide more target sites for its therapies (23, 24). Zuo et al. reported that *LINC00485* was located predominantly in the cytoplasm, we also found *LINC00485* was mainly located in the cytoplasm (25), indicating that *LINC00485* might act as a ceRNA by sponging corresponding miRNAs and thus indirectly regulate downstream mRNAs expression. After bioinformatics analysis of gene expression profiles, the lncRNA–miRNA interactions between *LINC00485* and elven miRNAs were screened out. Correlation analysis showed that the level of miR-205-5p, miR-141-3p, miR-338-3p, miR-372-3p, miR-373-3p, miR-216a-5p and miR-31-5p were not related to *LINC00485* in HCC. To further identify the correlation between *LINC00485* and miRNAs, we verified the expression levels of miR-214-3p, miR-195-5p, miR-93-5p and miR-424-5p in nine HCC-related datasets. The pooled results turned out, there were significant differences in the expression of the remaining four miRNAs, three of which (miR-214-3p, miR-195-5p and miR-424-5p) were shown to be down-regulated and miR-93-5p was up-regulated in HCC, it was reassuring to see that our pooled results are consistent with published articles (26–29).

Subsequently, this original study discovered that *LINC00485* may serve as a ceRNA competitively binding miR-214-3p, miR-195-5p, miR-93-5p and miR-424-5p, thus modulating ninety-four mRNAs expression. To better understand the regulation mechanism of this network, we established a PPI network and screened out ten hub genes (WEE1, RRM2, KIF23, E2F7, E2F2, E2F1, CHEK1, CEP55, CDC25A, CCNE1), and excluded miR-214-3p because no hub genes are associated with it. To the best of our knowledge, there is now accumulating evidences that have demonstrated the vital roles of hub genes in HCC (30, 31). In such a background, we performed correlation analysis, survival analysis and protein expression analysis to further identify potential key genes that are more relevant with HCC. Except CHEK1 and CEP55, eight genes were found significantly correlated with *LINC00485*, and the expression of WEE1 had no effect on the prognosis of HCC, KIF23, E2F7, CDC25A were excluded by HPA database. Functional enrichment and pathway analysis indicated that the remaining four hub genes play a crucial role in regulating some cancer-related biological processes, such as ‘Transcription factor binding’, ‘Regulation of cell cycle’ and ‘MicroRNAs in cancer’, this further confirming the critical roles of the four hub genes in HCC pathogenesis.

Notably, Numerous recent studies have established that these RNAs play significant roles in cancer progression. for example, Teng et al. demonstrated that downregulated miR-424-5p can facilitate tumor progression and angiogenesis in HCC by activating VEGFR-2 signaling pathway (28). Shi et al. also revealed that miRNA-93-5p promotes HCC progression *via* a microRNA-93-5p/MAP3K2/c-Jun positive feedback circuit (29). Furthermore, Huang et al. reported ANCCA/PRO2000 positively modulates E2F2 expression at both mRNA and protein levels, promoting cell cycle progression in HCC by enhancing cell proliferation (32). Yang et al. illustrated that Sorafenib can induce autophagy and growth inhibition in HCC cells by downregulating the expression of RRM2 (33). Overall, these findings and the results of our study consolidate the key functions of these RNAs in the progression and treatment of HCC. Lastly, we revealed a novel lncRNA–miRNA–mRNA regulatory network, which involved five pathway axes. This study might shed new light on the mechanism of *LINC00485* in HCC.

Our study did have some limitations that are worth noting. First, the sample size of the present study was relatively small, we couldn’t completely exclude that the statistical significance would be affected in some variable comparisons. Thus, a larger sample size is still indispensable to confirm the reliability of our results in future clinical research. Second, most of the enrolled cases were HBV-related HCC patients. Therefore, the clinical value of *LINC00485* in HCV-related HCC is still unknown, and Further studies need to address this point. Third, given that these five regulatory pathways were summarized based on bioinformatics and computational algorithms, the significance and practical functions of these five axes in HCC should be further investigated and more cellular experiments need to be conducted.

## Conclusion

Taken together, the present study indicated that overexpression of *LINC00485* could be used as potential diagnostic and prognostic biomarkers in HCC. Furthermore, we constructed a highly credible lncRNA–miRNA–mRNA regulatory network by integrated bioinformatics analysis, and each component of this ceRNA network is likely to play a significant role in regulating HCC progression. Our finding demonstrated the potential mechanism of *LINC00485* in HCC and revealed the bioprocess controlled by *LINC00485*.

## Data Availability Statement

The datasets presented in this study can be found in online repositories. The names of the repository/repositories and accession number(s) can be found in the article/**Supplementary Material**.

## Ethics Statement

The studies involving human participants were reviewed and approved by The Ethics Committee of Zhongnan Hospital of Wuhan University. The patients/participants provided their written informed consent to participate in this study.

## Author Contributions

XZ and JT conceived and designed the research. XZ and ZW performed the experiments. XZ, YF, and DH analyzed the data. XZ drafted the manuscript. ZW and FH revised the writing. All authors contributed to the article and approved the submitted version.

## Funding

This research was funded by the National Basic Research Program of China (2012CB720605) and Zhongnan Hospital of Wuhan University Science, Technology and Innovation Seed Fund (ZNPY2018067).

## Conflict of Interest

The authors declare that the research was conducted in the absence of any commercial or financial relationships that could be construed as a potential conflict of interest.

## Supplementary Material

Supplementary Table 1 Primers used in this study.

Supplementary Table 2 FASTA file of *LINC00485*.

Supplementary Table 3 Information of the antibodies used in IHC assay.

Supplementary Figure 1 The essential features of the nine datasets.

Supplementary Figure 2 The correlation between *LINC00485* and ten hub genes.

Supplementary Figure 3 The correlation between three miRNAs and ten hub genes.

Supplementary Figure 4 Survival analysis of eight hub genes.
