# Supplementary material for: Clinical Value and Underlying Mechanisms of Upregulated LINC00485 in Hepatocellular Carcinoma
Source: Front Oncol. 2021 Jul 5;11:654424. doi: 10.3389/fonc.2021.654424 (PMC8288074; doi:10.3389/fonc.2021.654424)
Supplement: Supplementary Table 1 — Primers used in this study. [file DataSheet_1.zip › Supplementary Materials/Supplementary Figure 1.pdf]

miRNA	First author	Year	Region	Data source	Platform	Number of case		Expression (mean±SD)	
						Cancer	Normal	Cancer	Normal
mir-214-3p	Su H	2012	China	GSE12717	GPL7274	9	6	8.298 [2.460]	9.549 [0.639]
	Sato F	2012	Japan	GSE21362	GPL10312	73	73	6.981 [1.545]	8.406 [0.587]
	Kim J	2013	South Korea	GSE39678	GPL15852	16	8	9.488 [0.742]	10.667 [0.530]
	Diaz G	2017	USA	GSE40744	GPL14613	9	19	3.307 [1.444]	4.333 [1.131]
	Morita K	2016	Japan	GSE41874	GPL7722	6	4	0.969 [0.224]	1.038 [0.197]
	Peng H	2019	USA	GSE64632	GPL18116	3	3	1.335 [0.314]	0.194 [0.048]
	Villanueva A	2017	Spain	GSE74618	GPL14613	218	10	1.678 [0.602]	2.113 [0.517]
	Xie Z	2018	China	GSE98269	GPL20712	3	3	7.165 [0.539]	8.225 [0.320]
	TCGA	2017	USA	TCGA	none	374	50	5.104 [2.084]	7.374 [0.785]
mir-195-5p	Su H	2012	China	GSE12717	GPL7274	10	6	10.483 [1.232]	12.798 [0.586]
	Sato F	2012	Japan	GSE21362	GPL10312	73	73	8.855 [1.187]	9.702 [0.510]
	Kim J	2013	South Korea	GSE39678	GPL15852	16	8	12.378 [1.125]	12.765 [0.360]
	Diaz G	2017	USA	GSE40744	GPL14613	9	19	2.148 [0.397]	2.626 [0.485]
	Morita K	2016	Japan	GSE41874	GPL7722	6	4	0.995 [0.239]	1.109 [0.227]
	Peng H	2019	USA	GSE64632	GPL18116	3	3	1.306 [1.169]	0.360 [0.366]
	Villanueva A	2017	Spain	GSE74618	GPL14613	218	10	1.383 [0.234]	1.394 [0.206]
	Xie Z	2018	China	GSE98269	GPL20712	3	3	9.211 [0.857]	10.216 [0.221]
	TCGA	2017	USA	TCGA	none	374	50	6.702 [1.479]	8.719 [0.839]
mir-93-5p	Su H	2012	China	GSE12717	GPL7274	10	6	12.543 [1.181]	10.514 [0.321]
	Sato F	2012	Japan	GSE21362	GPL10312	73	73	8.685 [0.860]	7.945 [0.537]
	Kim J	2013	South Korea	GSE39678	GPL15852	16	8	12.388 [0.704]	11.349 [0.125]
	Diaz G	2017	USA	GSE40744	GPL14613	9	19	6.292 [0.442]	5.134 [0.659]

	Morita K	2016	Japan	GSE41874	GPL7722	6	4	1.514 [0.473]	0.682 [0.038]
	Peng H	2019	USA	GSE64632	GPL18116	3	3	1.296 [0.371]	0.336 [0.128]
	Villanueva A	2017	Spain	GSE74618	GPL14613	218	10	1.867 [0.484]	1.602 [0.368]
	Xie Z	2018	China	GSE98269	GPL20712	3	3	8.825 [1.033]	8.213 [0.332]
	TCGA	2017	USA	TCGA	none	374	50	14.586 [0.897]	13.401 [0.688]
mir-424-5p	Su H	2012	China	GSE12717	GPL7274	10	6	8.667 [1.105]	12.107 [0.199]
	Sato F	2012	Japan	GSE21362	GPL10312	73	73	6.816 [1.527]	8.332 [0.814]
	Kim J	2013	South Korea	GSE39678	GPL15852	16	8	11.590 [0.677]	12.614 [0.428]
	Diaz G	2017	USA	GSE40744	GPL14613	9	19	5.707 [1.359]	7.856 [0.465]
	Morita K	2016	Japan	GSE41874	GPL7722	6	4	0.645 [0.087]	1.440 [0.407]
	Peng H	2019	USA	GSE64632	GPL18116	3	3	1.760 [1.595]	0.353 [0.250]
	Villanueva A	2017	Spain	GSE74618	GPL14613	218	10	2.122 [0.791]	3.114 [0.675]
	Xie Z	2018	China	GSE98269	GPL20712	3	3	8.201 [0.520]	9.712 [0.148]
	TCGA	2017	USA	TCGA	none	374	50	8.675 [1.242]	11.514 [1.027]
