# Supplementary material for: Clinical Value and Underlying Mechanisms of Upregulated LINC00485 in Hepatocellular Carcinoma
Source: Front Oncol. 2021 Jul 5;11:654424. doi: 10.3389/fonc.2021.654424 (PMC8288074; doi:10.3389/fonc.2021.654424)
Supplement: Supplementary Table 1 — Primers used in this study. [file DataSheet_1.zip › Supplementary Materials/Supplementary Table 1.docx]

**Supplementary Table 1.** Primers used in this study.

| **Primer name** |  | **Sequence (5’-3’)** |  |  |  |  |  |
| --- | --- | --- | --- | --- | --- | --- | --- |
| *LINC00485*-F  *LINC00485*-R  *GAPDH*-F  *GAPDH*-R |  | TCTCCATCACCCCCTGTTCT  TGAGCCGTTTTGTGGACTGT  GGTCTCCTCTGACTTCAACA  GTGAGGGTCTCTCTCTTCCT | | | | | |

Abbreviation: F: Forward; R: Reverse.
