# Supplementary material for: Clinical Value and Underlying Mechanisms of Upregulated LINC00485 in Hepatocellular Carcinoma
Source: Front Oncol. 2021 Jul 5;11:654424. doi: 10.3389/fonc.2021.654424 (PMC8288074; doi:10.3389/fonc.2021.654424)
Supplement: Supplementary Table 1 — Primers used in this study. [file DataSheet_1.zip › Supplementary Materials/Supplementary Table 2.docx]

**Supplementary Table 2**. FASTA file of *LINC00485*.

>ENST00000547179.1LINC00485-201cdna: lncRNA

ACTGCGCCCGAGAGGCAGCGCTCAGACAGCGGGGAGGAGGCGGCCGCGGAGCCCCCGGAGATGGAGGGTGAAGGCCTGCTTTCAAAGTATCCCACGTCGTTCAGCAAAGATTCGTCATTTAGAATTCAGACCTGCCAAGACCACGGATGCTTTGTCTCAGCTCGGATATCTTCATTTCTCCTTCTTACAGTTTCCTTGTTTCCGAGGAAAAACACAAGATCTTGACAACTGCAATCACCTGGTAACCAATCATGAATCTGGTATGGACCACTTTCACCCTGCACCCTGTCAGGCAGTTTCCATCTTCTCACAATCTTCACAAGAATCCTACAGAAACAAGACGTATCCCCCGATTTACTGATGATGATCCTTCAGCCCAGAGATGCACTCTCCATCGGCTGAACCCAACTGGAAGTCGGAGGGTAAAGGGCCCATGACTCAGTCCGTTCAGGCTGACCCGCCTGGGGCTCAGAGCATAACAGACAAGGGTGGGGAAGAAAAAAGAATGGACTTGGAGAAGCAAATGGGAAATATTCAGGCACTTCTGATCTCAGTTTGGCCAGTCGGCTCCCCTCTCTCTGACTGTAAACACAGAGATACAGAAACTGTCATTAGAAATGAGTAGTGTTAATGATCCATGACAGAAAGCTCGTGAATTTCTACTGCTGAGGAGGCTTGAGCTTTTCTGTTTTCATTCTTTCTACACATCGGGAGCGGAGCTCCCCAATGCCTTTCTGATACATCGCTACTTCTGCTTCACCTAATCAAAGGCACTTACTGCTGCTCAGAACCTCAGGCCTCCAACTGAACAATGAACAGGTTTTCTCTCCATCACCCCCTGTTCTCTAGTGTGAGTAGTTAGATTACTGCTTTTATGTCTGTCTTCCCAACTACAGTCCACAAAACGGCTCATATTGAACAGTAATACAAATAAAGAGAAAAAGGAGAGAAGACAAGAACAAAAAGTAATGAGTTTAGAAACTTGGAAAGTAGAAACTTCTTTGGTATGTTCCTGTTGAAAACCACTGGTCTCATCTTCCACTTTTTCACATTGCTGTTTCAGAGAAAAACTTAATTATACTTAATTATAGGGTTGTAATGTTTATAGGGTTGGTTATTCTGGTTAGCCAAATTTACTGGTTATCTGTTTAGAGCCTTTGAATTTTAATTCTAGCTGACACATTTGTTAAAACATTATTAGCTTACCTTTCTTCCTTAATGAGTACTGGATCTTCCCAGAGAAAAGGTTCCCAGTCATTTACCTGGAATTTTTTATACATATTTGCAGCTGTTCTTTGAGCTCCCTAAAGAGCATAATTAGAGACATAACATTCACTCAGATTTTCTGCCCTTCTTTGTAATAAGTCAATGAGCCAGTCATTTATTCCACAAATATTTGTTGATACCTACTGTGTGCCTGTCAGCAGTTCTCCAAGCAGGGGCTACAAAAATAAAAAGATTGTCTTTTAATGAGGAGCAACTTCGAGTCTTAGCTCTTGGTACTCCCTCTGTCTAGTAATATCCATTTTTATACTTAGTATTTTAATAAATAACTCTTCCTCAAATTATTTTAAAACACACAGTAACAGAAAAAATTATTTATTTATTCTACTTTCTCTTAATTACACCTGTGGGGAGCTTGGCGGGGCAGACTCTGGGTTTCCAGCCAAGGTCTTTCTGTCCACCTGGGGGCAGCAGACCCTCACACAGAAAAGTACCTCGAAGGAAGCAATTGGCTTTCTGAGCTCCTGGCTTTCAAACACAAGTCTTCCAAATTATGGACCCATGAATATTCTAAAGACTTATACTCGAAGGACGCTTCCAAAATCAAATAAATTTGGGTTGAACCAAGTTAAATAGGGTTTTAACCCTGCAGGACTTCTCACGATGTTTAATATACTTTTTTTTTTCTTTTCTTTCTTTCTTCGACAGAGTCTCGCTGTATTGCCAGACTGGAGTGCAGTGGCGTGATCTCGGCTCACTGCAACCTCCGCCTCCCAGGTTCAACTGATTCTCCTGCCTCAGCCTCCTGAGTAGCTGGGACTACAGGCGCCCACCACCACGCCCAGCTAATTTTTTTTTATTTTTAGTAGAGATGGGGTTTCACCATGCTGGCCAAGCTGGTCTCGAATTCCTGACCTCAGGTGATCCACCCGCGTTAGCCTCCCAAAGTGCTGACATTACAGGTGTGAGCCACTGCGCCGGGCCTAATATAGTTTTGTCGTACAAAACACGATGGCATACTTGCTCTGTGCAAAGTACATCACCCACAGTGGCCTATTTAAACTTGGCAACAGTCCTATGAGATAGGCACTAATTGTGTTCCCCATTTTACAAATGAGGAAACAGAGGCACGGAGCTTATAACTGGTGGCTGAGTTTCCGGTTGGGGCCATCTAGTTACAGAACTTGCATGCTTAATCATGACACCGTAATGCTTTGCAATGGGAGATTCCAAGAGGGGGAATTTGACATTCAGTGTTTCCCAAATTTATTTGGCTTTGGAACCCTTTTATTCCTTCTAATCAACATAGAGGACAAGTATGTAATGGGATACTCTGATAAATGCTTCAATAAAGCAATGGCTTTATGCCGCCCCGCTTTAAAAGATAACTAAAAATATAAGTCCACAGGCCCATCATATAATTGAACAGGTAACGACTGAATTTTGATTTGATGGAAAGACTCTTATCATATTAATCTAAGTTCTCAAAGTGAATGCCACTAAACCAAGACTATTCAGTGTATTTGAAGTATGTACAATACCAGTCCGAATTGGGACTTCTCAGGAAGAAAAAAGGGTATATGGCAGATGGGTAAGAATAGAAAGCCACAGGTCTAGCAGGGAAGCTAAATATGGTGTGGAATGTGTTGGGGAGATGGGAAAAATGCTCACCTCTTCATGGAAGTTCGAAAAGAAGGCTGAGATTCTAAATAATGCTGGAGTATTGCAGCTGGAACAAGCATGATAGCAATGGCCAACACAAGGCCTTTCTAGAATAAATCTAAAACAATTAAGAGATAATATGATAGCATTGGGAGATATACCTAATGCTAGATGACGAGTTAGTGGGTGCAGCGCACCAGCATGGCACATGTATACATATGTAACTAACCTGCACATTGTGCACATGTACCCTAAAACTTAAAGTATAATAATAATAAAAAGAAAAGAAAAAAAAAAGAGATAATATGCCAGGGGGTTGATGCCAGGTAATACTGATAGTTACCTTAAACAATG

TTTTCTCAACTTCAGTTTTTTACACACCAACTTCATGATTTTTTTCTATCAAGATACGAC

TACTATTATTTATCTAGCGTTTTTTAAATTGTCTTATATCTTTGCTTAAATTTATTTTTTTAAACATTAATATCTGAGAATTCATCATTTTGAAGGAATAATTATATTGTTCTAATATATAAATTTTAAAGTTAATATCTTTATTCCATCTAAAGTGGTATGTGTACTATTCTTTGGAAATGATTTTCTAAAGGATTAAAAACTTGCCAGTGTTGGAAAAAAAAGGAAACAGGAAAATTAAGACTGAGTTAAAGAAGGTGAAAACAAAATTGAAGAATAAGACTAAAGGTTAGACATGAA

ATAGATCTGTCTCTAATTAGAGGTGCAATGTCAAATTTTTTTGTAGATCGTTCTAAAAAT

ATCACTTGTTCTTTTGCTTGTATTTTAATTCAATGATTAAATAAACAGATGACCTAGTTT

CA
