# Supplementary material for: Clinical Value and Underlying Mechanisms of Upregulated LINC00485 in Hepatocellular Carcinoma
Source: Front Oncol. 2021 Jul 5;11:654424. doi: 10.3389/fonc.2021.654424 (PMC8288074; doi:10.3389/fonc.2021.654424)
Supplement: Supplementary Table 1 — Primers used in this study. [file DataSheet_1.zip › Supplementary Materials/Supplementary Table 3.docx]

**Supplementary Table 3.** Information of the antibodies used in IHC assay.

1. **Anti-RRM2 antibody** (Rabbit polyclonal to RRM2), Atlas Antibodies Cat#: HPA056994, the antibody was validated by the company using Enhanced antibody validation method

(Orthogonal validation) with the validation score of “Enhanced”.

please refer to: https://www.proteinatlas.org/ENSG00000171848-RRM2/antibody.

5. **Anti-KIF23 antibody** (Rabbit polyclonal to KIF23), [Santa Cruz Biotechnology Cat#: sc-867,](http://antibodyregistry.org/search.php?q=AB_631959) the antibody was validated by the company using Standard antibody validation method

(literature conformity and RNA consistency) with the validation score of “Uncertain”.

please refer to: https://www.proteinatlas.org/ENSG00000137807-KIF23/antibody.

8. **Anti-E2F2 antibody** (Mouse monoclonal to E2F2), Santa Cruz Biotechnology Cat#: sc-9967,

the antibody was validated by the company using Standard antibody validation method

(literature conformity and RNA consistency) with the validation score of “Uncertain”.

please refer to: <https://www.proteinatlas.org/ENSG00000007968-E2F2/antibody>.

7. **Anti-E2F1 antibody** (Rabbit monoclonal to E2F1), [Origene](http://www.origene.com/) Antibody CAB019308, the

antibody was validated by the company using Standard antibody validation method (literature

conformity and RNA consistency) with the validation score of “Approved”.

please refer to: https://www.proteinatlas.org/ENSG00000101412-E2F1/antibody.

6. **Anti-CCNE1 antibody** (Rabbit polyclonal to CCNE1), [Atlas Antibodies Cat#: HPA018169](http://antibodyregistry.org/search.php?q=AB_1847384), the antibody was validated by the company using Standard antibody validation method (literature

conformity and RNA consistency) with the validation score of “Approved”.

please refer to: https://www.proteinatlas.org/ENSG00000105173-CCNE1/antibody.

9. **Anti-AFP antibody** (Mouse monoclonal to AFP), [R&D Systems](http://www.rndsystems.com/) Antibody CAB025339, the antibody was validated by the company using Standard antibody validation method (literature

conformity and RNA consistency) with the validation score of “Approved”.

please refer to: https://www.proteinatlas.org/ENSG00000081051-AFP/antibody.
