# Supplementary figures and images for: Clinical Value and Underlying Mechanisms of Upregulated LINC00485 in Hepatocellular Carcinoma
Source: Front Oncol. 2021 Jul 5;11:654424. doi: 10.3389/fonc.2021.654424 (PMC8288074; doi:10.3389/fonc.2021.654424)

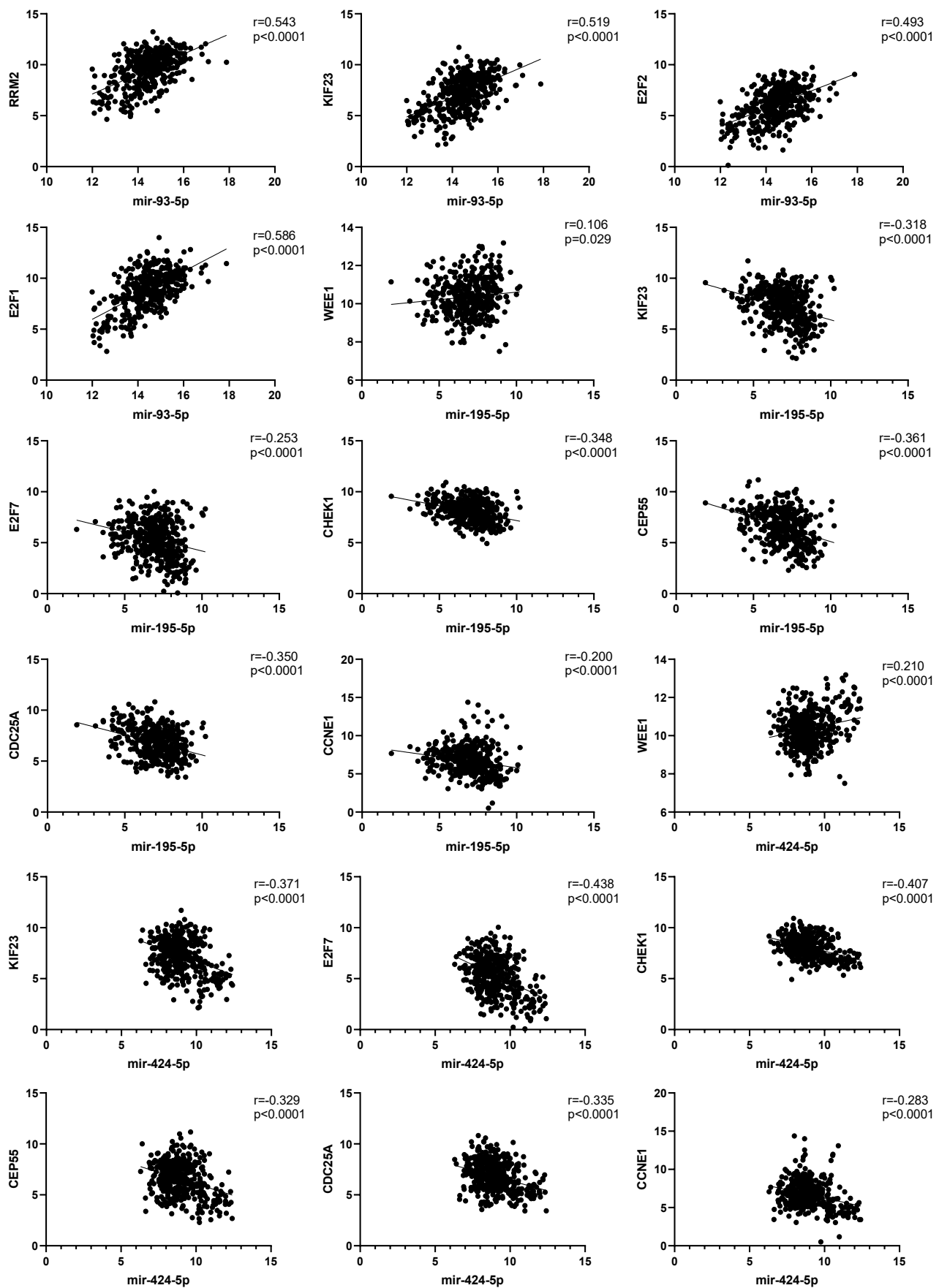

Supplement: Supplementary Table 1 — Primers used in this study. [file DataSheet_1.zip › Supplementary Materials/Supplementary Figure 2.pdf]

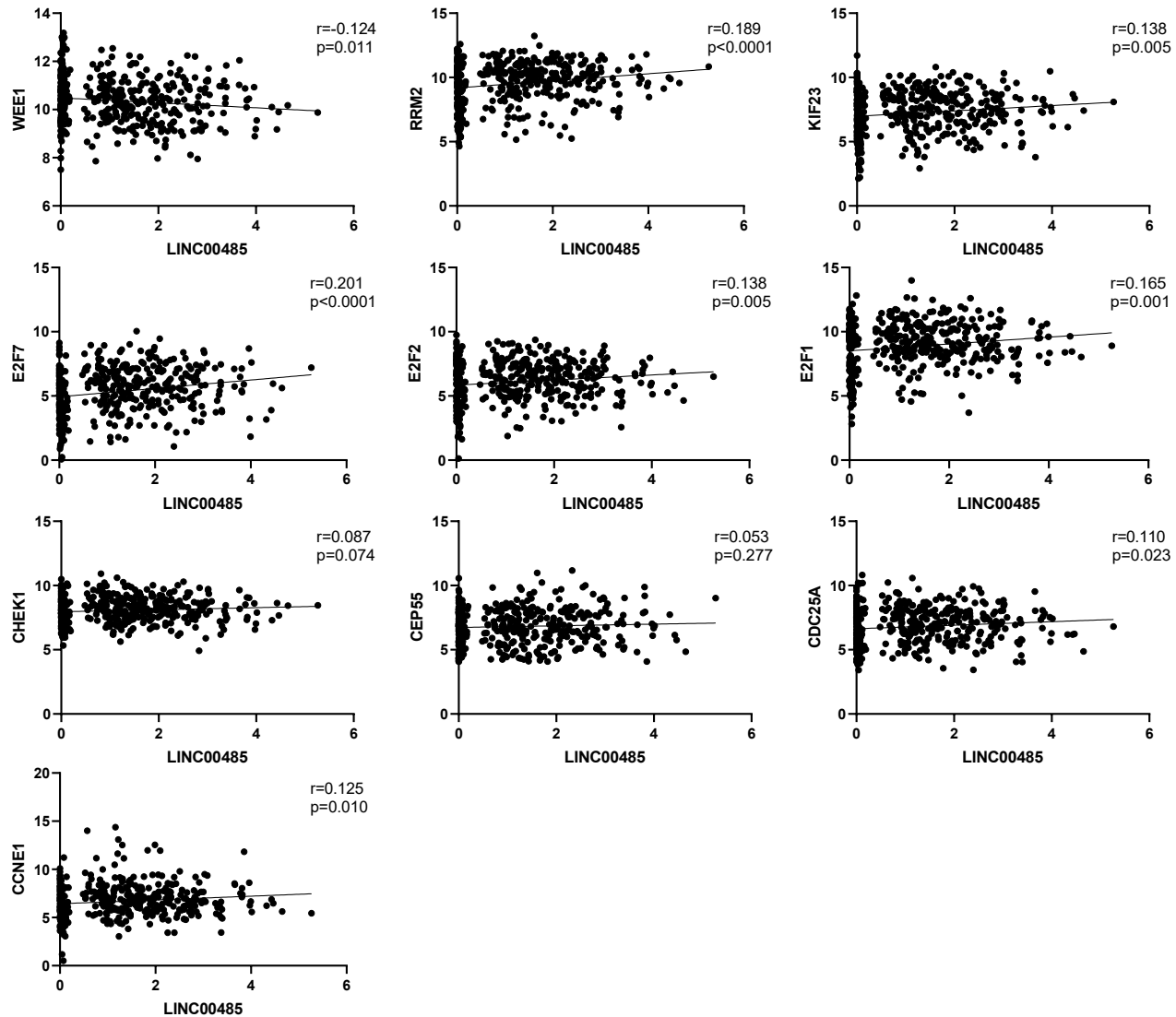

Supplement: Supplementary Table 1 — Primers used in this study. [file DataSheet_1.zip › Supplementary Materials/Supplementary Figure 3.pdf]

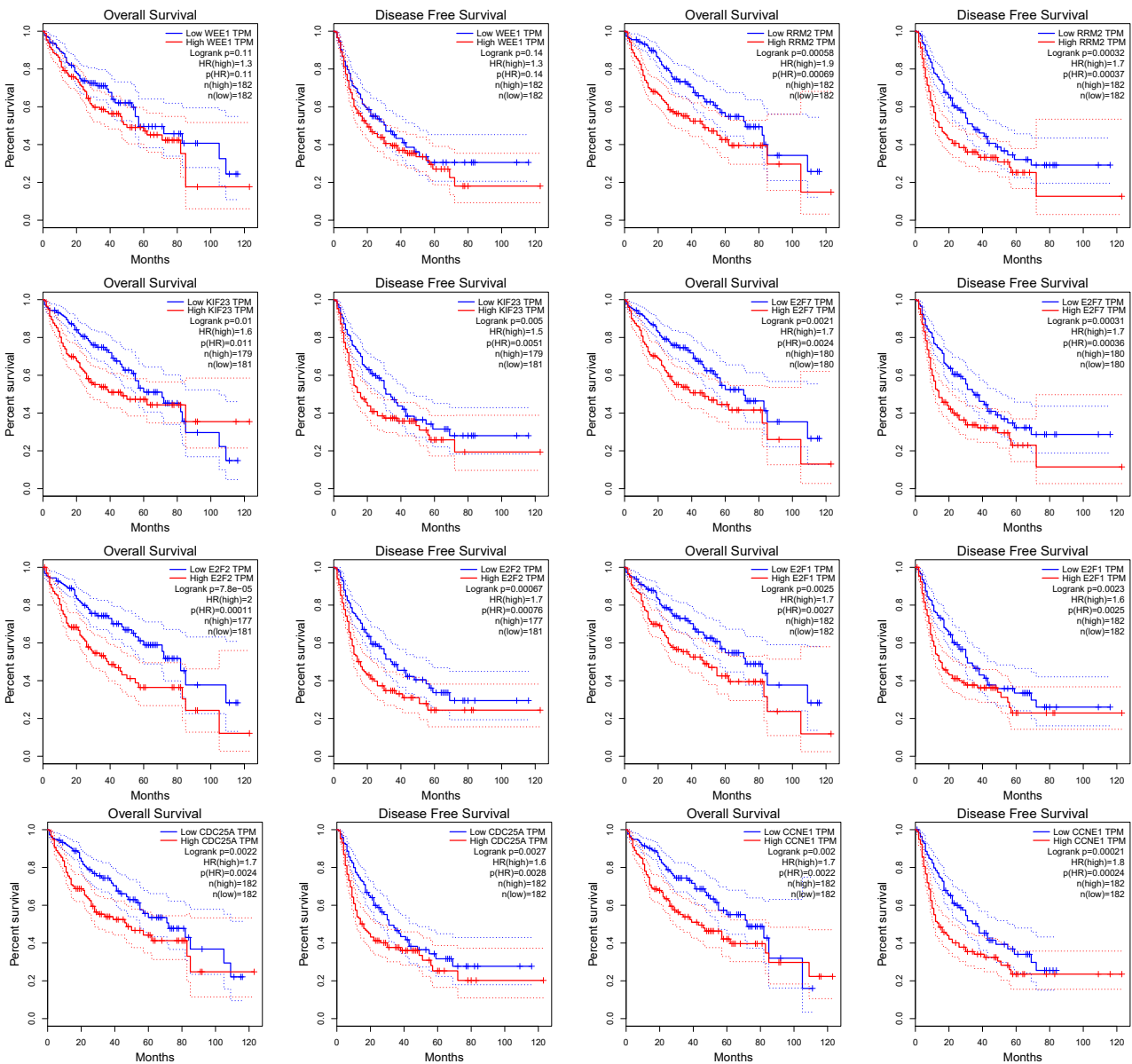

Supplement: Supplementary Table 1 — Primers used in this study. [file DataSheet_1.zip › Supplementary Materials/Supplementary Figure 4.pdf]
